# The mitochondrial genome of booklouse, *Liposcelis sculptilis* (Psocoptera: Liposcelididae) and the evolutionary timescale of *Liposcelis*

**DOI:** 10.1038/srep30660

**Published:** 2016-07-29

**Authors:** Yan Shi, Qing Chu, Dan-Dan Wei, Yuan-Jian Qiu, Feng Shang, Wei Dou, Jin-Jun Wang

**Affiliations:** 1 Key Laboratory of Entomology and Pest Control Engineering, College of Plant Protection, Southwest University, Chongqing 400716, China

## Abstract

Bilateral animals are featured by an extremely compact mitochondrial (mt) genome with 37 genes on a single circular chromosome. To date, the complete mt genome has only been determined for four species of *Liposcelis*, a genus with economic importance, including *L. entomophila*, *L. decolor*, *L. bostrychophila*, and *L. paeta*. They belong to A, B, or D group of *Liposcelis*, respectively. Unlike most bilateral animals, *L. bostrychophila*, *L. entomophila* and *L. paeta* have a bitipartite mt genome with genes on two chromosomes. However, the mt genome of *L. decolor* has the typical mt chromosome of bilateral animals. Here, we sequenced the mt genome of *L. sculptilis*, and identified 35 genes, which were on a single chromosome. The mt genome fragmentation is not shared by the D group of *Liposcelis* and the single chromosome of *L. sculptilis* differed from those of booklice known in gene content and gene arrangement. We inferred that different evolutionary patterns and rate existed in *Liposcelis*. Further, we reconstructed the evolutionary history of 21 psocodean taxa with phylogenetic analyses, which suggested that Liposcelididae and Phthiraptera have evolved 134 Ma and the sucking lice diversified in the Late Cretaceous.

During the last two decades, the booklice of the genus *Liposcelis* have emerged as serious pests of stored commodities worldwide^1^. Moreover, many microorganisms, such as rickettsial species have been found inside and on outside surfaces of booklice bodies, and these could be transferred to humans thereby posing a threat to human health^2^,^3^.

Recently, several types of atypical mitochondrial (mt) genome organization have been reported in psocodean insects (superorder Psocodea). Psocodea contains two orders of insects: Psocoptera (booklice and barklice) and Phthiraptera (chewing and sucking lice). The mt genomes of human lice, *Pediculus humanus*, *P. capitis* and *Pthirus pubis*, consist of 14 to 20 mini-chromosomes, each is 1.8 to 4 kb in size and contains one to five genes^4^,^5^, while *nad4* was unidentified in *Pthirus pubis*^5^. *Haematomyzus elephantis* from the suborder Rhynchophthirina was sequenced with ten minichromosomes^6^, and *nad2* and three tRNA were unidentified. The chewing louse, *Coloceras* sp., has a typical mitochondrial chromosome with 37 genes and a circular mt DNA molecule that is approximately half the size of the typical mt chromosome^7^. The booklice, *L. bostrychophila*, has a bipartite mt genome with two chromosomes: one chromosome is <8 kb in size and has 16 genes and the other is <8.5 kb in size and has 22 genes^8^. The mt genome of *L. decolor* has the typical mitochondrial chromosome of bilateral animals, 14,405 bp long with 37 genes (13 PCGs, 22 tRNAs and 2 rRNAs). For *L. entomophila* and *L. paeta*, one mitochondrial chromosome has most of the mt genes whereas the other chromosome has largely pseudogenes and non-coding regions^1^. Intriguingly, *nad4L* was absent in *L. entomophila*^1^. *L. entomophila* and *L. paeta* differ substantially from each other and from *L. bostrychophila* in gene content and gene arrangement in their mt chromosomes, although they have evolved multipartite mt genomes. However, a recent influential study has discovered maternally transmitted sex ratio distortion in *L*. nr. *bostrychophila* that is associated with extraordinarily divergent mitochondria. Sequencing the mt genomes of distorter and normal individuals proved to be quite a surprise. Not only were they incredibly divergent, they also had radically different gene order and genome structure. Both distorter and normal individuals had multipartite mt genomes, consisting of at least five and seven minicircles, respectively^9^. Moreover, *L. decolor*, the arrangement of genes differs substantially from that observed in *Liposcelis* species and other insects^10^. The genus *Liposcelis* was classified into four groups (A, B, C and D) and has 126 known species worldwide^11^. The booklouse, *L. sculptilis,* investigated in the current study, belongs to D group. In present, *L. bostrychophila* and *L. paeta* of D group, bipartite mt genome with two chromosomes, have been sequenced. Do all the species from D group have the two mitochondrial chromosomes structure? Based on the hypothesis, we sequenced the mt genome of *L. sculptilis*.

Despite being classified as Psocoptera, the suborder Psocomorpha is phylogenetically more closely related to the Phthiraptera than to the another suborder Trogiomorpha^1^,^10^,^12^. Because the fossil record for lice is poor^13^, there was only few studies that attempted to the evolutionary history among Psocodea suborders and among Anoplura families^14^,^15^. In addition, they only used the portion of the *cox1*, *18S* and *EF-1* gene sequences to reconstruct the phylogenetic relationship and evolutionary history^14^,^15^. This study is the first attempt to use mt genome to elucidate the evolutionary history of this unique group.

## Results

### Mitochondrial genome of *Liposcelis sculptilis*

**Figure 1:**
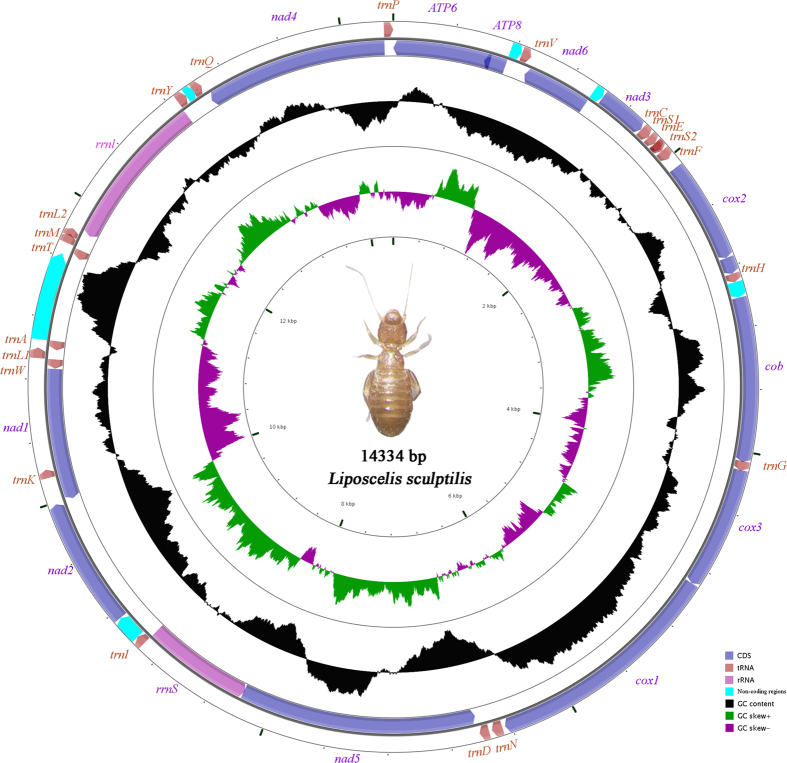
Mitochondrial genomes of *Liposcelis sculptilis.* Circular maps were drawn with CGView^55^. Arrows indicate the orientation of gene transcription. Protein-coding genes are shown as blue arrows, rRNA genes as purple arrows, tRNA genes as brown arrows and non-coding regions as light green. Abbreviations of gene names are: *atp6* and *atp8* for ATP synthase subunits 6 and 8, *cox1*-*3* for cytochrome oxidase subunits 1-3, *cob* for cytochrome b, *nad1*-*6* for NADH dehydrogenase subunits 1-6, *rrnL* and *rrnS* for large and small rRNA subunits. tRNA genes are indicated with their one-letter corresponding amino acids; the two tRNA genes for leucine and serine have different anticodons: L1 (anticodon TAG), L2 (TAA), S1 (TCT) and S2 (TGA). The GC content is plotted using a black sliding window, as the deviation from the average GC content of the entire sequence. GC-skew is plotted as the deviation from the average GC-skew of the entire sequence. The inner cycle indicates the location of genes in the mitochondrial genome.

The mt genome of *L. sculptilis* has one typical circular chromosome (Fig. 1), unlike the other two species from D group of booklice, including *L. bostrychophila* and *L. paeta*. The size and the circular organization of the mt chromosome of *L. sculptilis* was confirmed by two overlapping PCR amplicons, 9.1 kb (S9–S10 from *cob* to *rrnL*) and 5.5 kb in size (S11–S12 from *rrnL* to *cob*), respectively (Fig. 1, S1; Table 1). The two amplicons overlapped by 42 bp in *cob* and 74 bp in *rrnL*. We found 35 of the 37 genes typically for bilateral animals in *L. sculptilis* mt genome, and it contains 12 protein-coding genes (*atp6*, *atp8*, *cob*, *cox1*-*cox3*, *nad1*-*nad6*), two rRNA genes (*rrnL*, *rrnS*) and 21 tRNA genes.

### The variation structure of tRNA in *L. sculptili*

**Figure 2:**
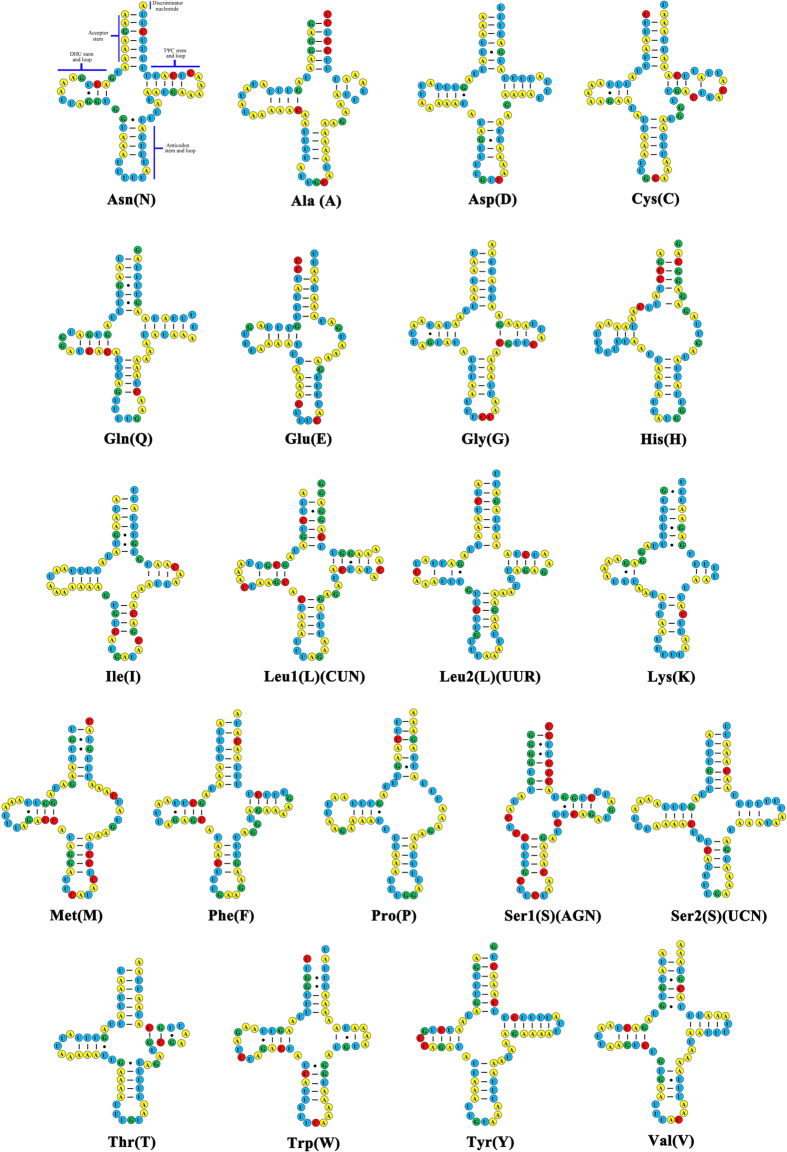
Putative secondary structures of the 21 tRNA genes identified in the mitochondrial genome of *L. sculptilis.* Bars indicate Watson-Crick base pairings, and dots between G and U pairs mark canonical base pairings in RNA.

Most tRNAs of the *L. sculptilis* have the typical clover leaf shaped secondary structure. However, five tRNAs have atypical structures. For TΨC arm and DHU arm, tRNA-His, tRNA-Glu, tRNA-Met, and tRNA-Pro lack the TΨC arm, and tRNA-Ser1 lack DHU arm in the *L. sculptilis* (Fig. 2). In most insects, tRNA-Ser1 also lacks the DHU arm. In terms of anti-codon, 16 tRNAs use common antisense codons, however, five tRNAs (tRNA-Asn, tRNA-His, tRNA-Ile, tRNA-Lys, tRNA-Ser1) use uncommon antisense codons. UUU, AUG, GAU, UUU, and UCU replaced the traditional codons GUU, GUG, AAU, CUU, and GCU, respectively. Secondary structure of 19 tRNAs, 62 mismatches, have base mismatch. There are 38 G-U mismatches, five U-C mismatches, six G-A mismatches, four U-U mismatches, four A-A mismatches, and four A-C mismatches.

### Mitochondrial gene codon usage of the booklice

**Figure 3:**
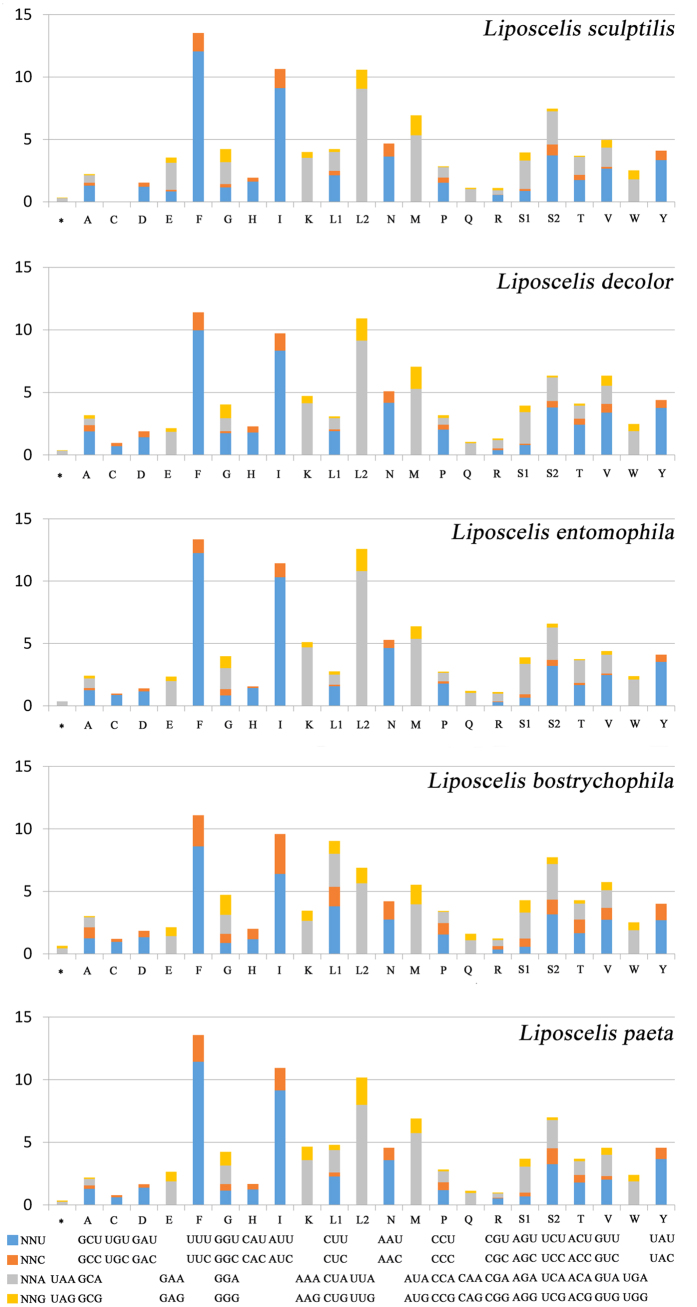
Relative synonymous codon usage (RSCU) for protein coding genes of five booklice. Abbreviations of tRNA genes are according to the single letter according to the IPUC-IUB one-letter amino acid codes.

To date, three mt genomes of *L. bostrychophila, L. sculptilis* and *L. paeta* from D group of *Liposcelis* have been sequenced. The A + T content of the *L. sculptilis* mt genome was 76.5%, which was higher than *L. paeta* (75.23%) and *L. bostrychophila* (68.63%), respectively. The higher A + T content of *L. sculptilis* was present in all regions, both genes and noncoding regions. Phe (F), Ile (I) and Leu (L) are most frequently used and the frequency used of the two codons of Leu are obviously different in the five *Liposcelis* species (Fig. 3). In these booklice, the frequency of L2 (UUA and UUG) was significantly higher than that of L1(CUN). However, this result was just the opposite in *L. bostrychophila* (Fig. 3). Moreover, the difference of A + T content between the five booklice were also reflected further in the codon usage: the relative synonymous codon usages (RSCU) of the five booklice showed that *L. sculptilis* used more NNA and NNT codon than *L. paeta* and *L. bostrychophila*. The nucleotide composition of mt genome is usually conserved within a group; however, it varied among *L. sculptili*, *L. paeta* and *L. bostrychophila*. This variation might be related to mt genome fragmentation, because all of the psocodean fragmented mt genomes have a lower A + T content^10^.

In total of 63 mt protein-coding genes in five *Liposcelis* species, the traditional codon ATN (60) were used as start codon. However, the *cox1* of *L. decolor* and the *cox3* of *L. paeta* use TTG and the *atp8* of *L. bostrychophila* use GTG as start codon. In terms of the stop codon, the codon TAA and TAG were used as stop codon in 61 genes. However, incomplete stop codon was used for the *nad1* of *L. bostrychophila* and the *nad4L* of *L. paeta,* respectively (Table 2).

### Mitochondrial gene order of the *Liposcelis*

**Figure 4:**
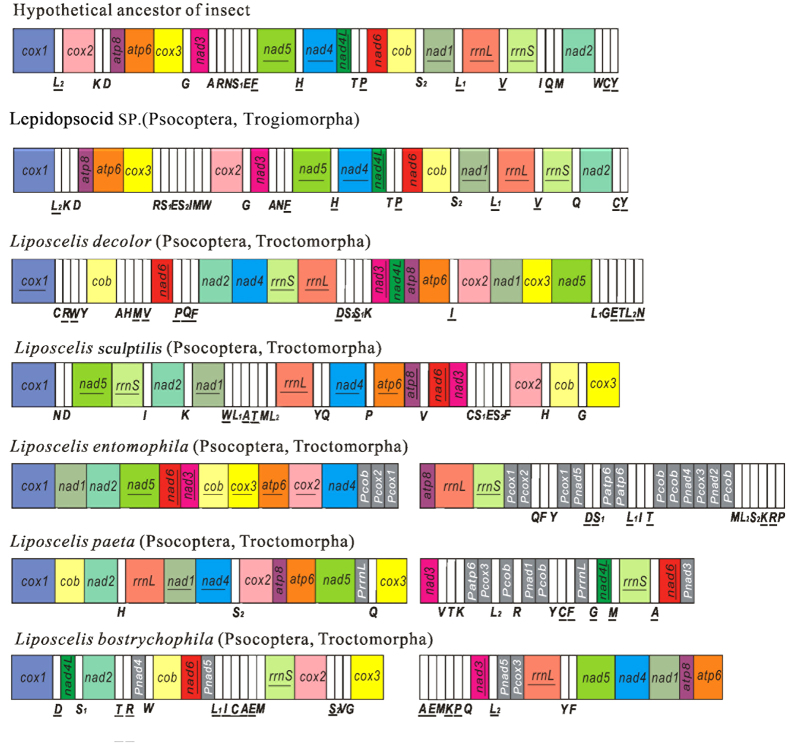
Arrangement of mitochondrial genes in *Liposcelis* and the hypothetical ancestor of the arthropods. Circular genomes have been arbitrarily linearized for ease of comparison. Gene names are the standard abbreviations used in the present study. tRNA genes are designated by the single letter according to the IPUC-IUB one-letter amino acid codes. Genes which are underlined are encoded on the opposite strand to the majority of genes in that particular genome. Gray, and white boxes represent pseudogenes, and transfer RNA genes, respectively. The boxes in 15 colors represent 13 protein coding genes and 2 ribosomal RNA genes.

In the present work, the five *Liposcelis* species belong to three groups (A, B, and D). *L. bostrychophila*, *L. paeta* and *L. sculptilis* belong to D group, and their phylogenetic relationship has been demonstrated in our phylogenetic trees. However, they have huge differences in the size, gene content, gene order and architecture of the mt genome. The mt gene arrangement in *Liposcelis* differs substantially from that of the hypothetical ancestor of insects and from that of the barklice (Fig. 4). With the exception of *L. entomophila*, there is only *atp8*-*atp6* gene block shared between booklice and barklice, even though these five booklice belong to the same genus. In D group of *Liposcelis*, we found that *L. bostrychophila*, *L. paeta* and *L. sculptilis* shared two gene blocks: *atp8*-*atp6*, *cox3*-*cox1* (Fig. 4).

### Phylogenetic relationships and divergence times of the Psocodea

**Figure 5:**
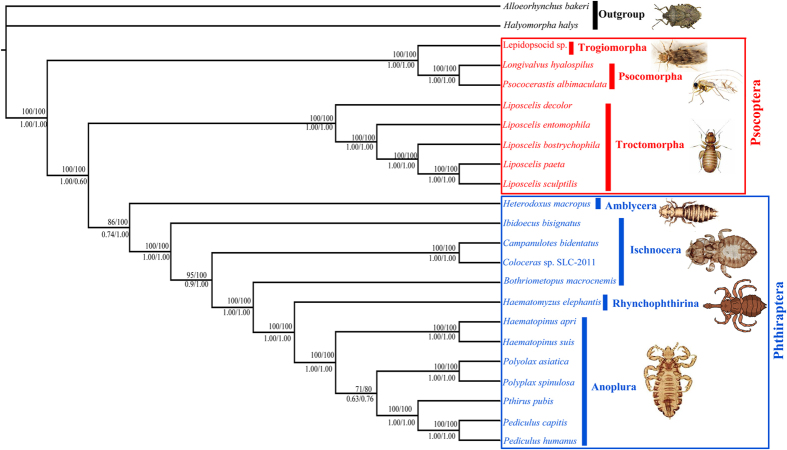
Phylogenetic relationships among major lineages of the Psocodea inferred from mitochondrial genome sequences. Numbers close to the branching points are ML bootstrap support values (above) and Bayesian posterior probabilities (below) in percentages; only support above 50% is shown. Numbers from left to right are from PCG123R and AA alignments respectively.

**Figure 6:**
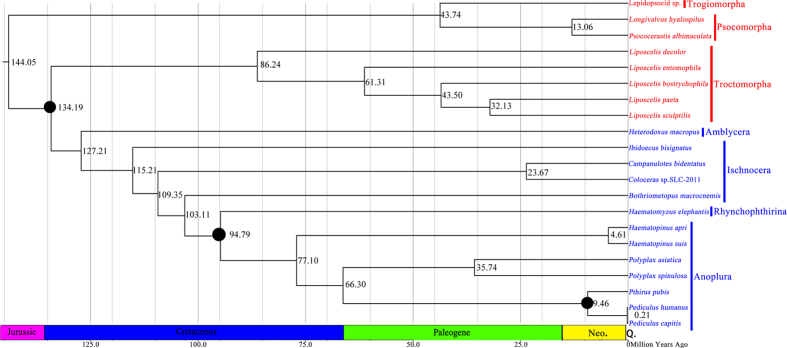
Chronogram for the Psocodea. Chronogram is the Bayesian topology resulting from analysis of the 11 genes (*cox1*, *cox2*, *cox3*, *atp6*, *cob*, *nad1*, *nad3*, *nad5*, *nad6*, *rrnS* and *rrnL*) data set in BEAST^53^. Divergence times were estimated using three calibrations (The potential ancient origin of lice considering the recently described 100–145 Ma book louse fossil; 94–101 Ma for the split between Rhynchophthirina and Anoplura; this ancestral to all human lice has been stable for at least 7 Ma), indicated by filled circles at nodes.

We tested the phylogenetic relationships among the major lineages of the Psocodea together with the mt genome sequences of *L. sculptilis*, and twenty other psocodean species (Fig. 5). Based on the two different datasets, we recovered two major clades in the Psocodea with strong support values regardless the dataset and the method we used: 1) species of barklice in the suborders Psocomorpha were clustered together; 2) the booklice formed a clade with the parasitic lice (Fig. 5). The parasitic lice (Phthiraptera) are monophyletic with strong support; however, within the parasitic lice, the suborder Ischnocera is paraphyletic and the sister-group relationship between Anoplura and Rhyncophthirina was also strongly supported. For the divergence times of the Psocodea, our results showed that Liposcelididae and Phthiraptera have evolved 134 Ma and the sucking lice diversified in the Late Cretaceous, approximately 77 Ma. Moreover, the Anoplura and Rhynchophthirina have evolved 94 Ma (Fig. 6 and further details are shown in Fig. S2).

## Discussion

### The unidentified genes of the Psocodea

Mt genomes of bilaterians typically contain two rRNA, 22 tRNA, and 13 protein-coding genes (PCGs) and a control region on a single circular chromosome, with ~16 kb in size^16^,^17^. This typical mt genome organization is conserved among most of the bilateral animals known from worms to insects, fish, and humans^18^,^19^,^20^,^21^. On the other hand, however, deviation from the typical mt genome organization has occurred in many bilateral animals. For instance, most nematodes lose *atp8* gene and thus only have 36 mitochondrial genes^22^,^23^, as well as in a tree frog species in which the *nad5* gene is apparently missing^24^. So far, the reported species of the Psocodea, some PCG have not been identified in *L. entomophila, L. sculptilis, Pthirus pubis,* and *Haematomyzus elephantis*, i.e., *nad4* and *nad2* have not been found in the pubic louse and elephant louse, respectively^5^,^6^. Additional, *nad4L* has not been found in *L. entomophila*^1^. Intriguingly, we also did not find *nad4L* in *L. sculptilis*. However, *nad4L* is present in the other three species of *Liposcelis* (*L. bostrychophila, L. decolor*, and *L. paeta*)^1^,^8^,^10^. There are many possibilities for lacking mitochondrial genes. Firstly, due to sequencing technology and sequencing method, a mini-chromosome contained *nad4L* (or *nad4* or *nad2*) gene, which is not identified in *L. sculptilis* and *L. entomophila* (or *Pthirus pubis* and *Haematomyzus elephantis*). For example, the mt genome architecture in some thrips exhibit extreme chromosome size asymmetry that only *nad6* and tRNA-Cys are on the 0.92 kb mini-circle chromosome^25^. Secondly, these missing genes are functionally replaced by nuclear genes or normally transferred to the nuclear genome. For instance, mt *cox2* introgression into the nuclear genome has been reported^26^. Thirdly, the lacking genes have been lost in the long process of evolution. In fact, as some protein-coding genes of mt genes are essential in mt respiration and adenosine triphosphate production^27^, the loss of these genes would present serious metabolic challenges to cells. We inferred that the lacking of *nad4L* gene in *L. sculptilis*, more likely due to the first or the second possibilities.

For the unidentified tRNA genes in the *Liposcelis*, eight tRNA genes have not been found in mt genome of *L. entomophila* or *L. paeta*. Additionally, two of 22 typical tRNA genes were also not found in *L. bostrychophila*^1^,^8^, and one tRNA was unidentified in the *L. sculptilis*. Lacking of tRNA genes is more diverse, and, for example, has been reported in one gekkonid^28^, in one caecilian amphibian^29^, and in isopod crustacean^30^. Moreover, among lower metazoans, a massive loss of tRNA was reported in cnidarians^31^. Altogether, it is unclear whether in these cases the gene loss is due to gene transfer to the nuclear genome or reflects loss of the protein function^32^.

### The mitochondrial genome strand asymmetry and structure of the *Liposcelis*

Strand asymmetry is reflected by AT skew and GC skew. Positive AT skew values indicate more A than T on the target strand, and positive GC skew values indicate more G than C, and vice versa^33^. In terms of *L. sculptilis*, the A + T content is 76.5%, which was lower than the *L. entomophila* (78.6%) in *Liposcelis*. The AT skew is 0.018 and the CG skew is 0.047, and these results indicated a low degree of strand asymmetry of the base composition in the *L. sculptilis* mt genome. Generally, insect mt genomes, in terms of the structure, gene order, gene content, are very stable, however, this rule does not apply in the genus of *Liposcelis*. From the results of our studies in five *Liposcelis* species, the tremendous differences in these features were existed (Figs 3 and 4). Such differences among mt genomes, were first discovered within a genus in insects. Even some of the gene blocks are stable within a genus of *Anoplura,* which have highly fragmented mt genomes and violently gene rearrangement. For instance, the sucking lice, *Pediculus humanus* and *P. capitis*, have fragmented mt genomes with 20 minichromosomes, but the gene order of protein-coding genes and gene content, were identical across the corresponding minichromosomes^4^,^5^. The same example also appeared between the pig lice, *Haematopinus apri* and *H. suis*^34^. The phylogenetic relationships between booklice and Phthiraptera are close from gene rearrangement. However, three barklices (Lepidopsocid sp., *Psococerastis albimaculata,* and *Longivalvus hyalospilus*), have relatively conservative gene order^12^,^35^, also belong to Psocoptera. They shared lots of gene blocks. For the convenience and accuracy of communication, our previous studies have introduced the concept of “mitochondrial karyotype” or “mitochondrial genome karyotypes” for describing the violent variation of mt genomes in sucking lice and booklice^6^,^36^.

### Phylogenetic relationships among major lineages of the *Liposcelis* inferred from mitochondrial genome sequences

The phylogenetic tree showed a close relationship between the booklice (*Liposcelis*) and parasitic lice (sucking lice). Therefore, the order Psocoptera was paraphyletic, and this result was consistent with previous studies^13^,^17^,^18^,^19^. Mt gene rearrangement has been substantially faster in the lineage leading to the booklice and the parasitic lice than in the lineage leading to the barklice^12^. Lifestyle change of booklice appears to be associated with the contrasting rates in mt gene rearrangements between the two clades of the Psocodea^37^,^38^,^39^.

Within the genus *Liposcelis*, *L. paeta* and *L. sculptilis* formed a clade and then clustered with *L. bostrychophila*. These three species, from group D^40^, were most closely related to *L. entomophila* (group A). The current classification of genus *Liposcelis* included four groups (A, B, C, and D)^11^,^41^, which have big difference in morphological, physiology and molecular biology aspects^41^,^42^. Although the five *Liposcelis* species are different from each other in mt genome organization, they were clustered together with strong support in the phylogenetic tree. Then, these results suggest unusually fast evolution in mt genome organization in the booklice of the genus *Liposcelis*.

### Estimation of divergence times

Fossil calibrations for lice are lacking^13^,^15^. Based on a fossil calibration, previous study indicated that parasitic lice and booklice minimally have diverged 100 Ma^13^. For a basal calibration representing the split between Rhynchophthirina and Anoplura, we chose the time of basal diversification in placental mammals in a previous study^43^, because we believed it reasonable that sucking lice could not have diversified until they had appropriate hosts to colonize^43^,^44^. It has been determined that time of basal diversification in placental mammals was 94–109 Ma; therefore, a calibration with a mean of 101 (and standard deviation of 3.5) was used to represent the basal split between the Rhychophthirina and Anoplura^14^,^43^. Additionally, some studies have documented the most recent common ancestor of human lice (*Pediculus humanus*, *Pediculus capitis*, and *Pthirus pubis*) has been stable for at least 7 Ma^5^. Totally, these three calibration points were used in combination, as well as individually, to cross-check the other calibration points, in the divergence dating analyses.

Our analyses indicated that the most recent common ancestor of Liposcelididae and Phthiraptera has been diversified in Late Jurassic to Early Cretaceous, approximately 134 Ma (Fig. 6). The estimated divergence time of Liposcelididae and Phthiraptera was largely consistent with a recent estimates^45^. Moreover, the results, sucking lice diversified in the Late Cretaceous, approximately 77 Ma; Anoplura and Rhynchophthirina have evolved 94 Ma (Fig. 6), were also consistent with a previous study^14^. However, another previous study reported that Liposcelididae and Amblycera were sister group and the Ischnocera was monophly, was inconsistent with the most recent research^1^,^8^,^10^,^12^,^15^.

## Materials and Methods

### Sample collection, DNA extraction and amplification of mitochondrial genome

*L. sculptilis* was collected at a grain storage in Gansu Province, China, and identified according to their morphological characteristics^11^,^44^. Total genomic DNA was extracted from ~300 booklouse specimens (20 mg) using a TIANamp Genomic DNA Kit (Tiangen Biotech, Beijing, China) and stored at −20 °C. Partial sequences of *cox1*, *cob*, *nad5*, and *rrnL* of *L. sculptilis* were amplified initially by PCR with primer pairs UEA3-UEA8 (1016 bp), CBF1-CBR1 (481 bp), N5-F100-N5-R100 (311 bp) and 16Sar-16Sbr (513 bp), respectively (Table 1). Four pairs of primers, S1-S2, S3-S4, S5-S6 and S7-S8, were designed from *cox1*, *cob*, *nad5*, and *rrnL* (Table 1). Four overlapping fragments were amplified by long PCR with S1-S2, S3-S4, S5-S6 and S7-S8, sequenced and assembled into contigs with SeqMan (DNAStar). To verify *L. sculptilis* chromosome and avoid the mistake might be caused by primers (S9-S10 and S11-S12) at *cob* and *rrnL*, a 9,005 bp fragment and a 5,631 bp fragment were amplified additionally with primers S9-S10 and S11-S12 (Table 1).

LA Taq (5 U/μL, Takara) was used in long PCRs to amplify overlapping fragments. Each long PCR reaction is 25 μL in volume, containing 1.0 μL each of forward primer (10 μM) and reverse primer (10 μM), 4.0 μL of dNTPs mix (each 2.5 mM), 1.0 μL of template DNA, 2.5 μL MgCl_2_ (25 mM), 2.5 μL of 10 ×  LA PCR reaction buffer II, 12.75 μL ddH2O and 0.25 μL LA Taq DNA polymerase (5 U/μL, Takara). All reactions were carried out using C1000^TM^ thermal cyclers (Bio-RAD, Hercules, CA, USA) with the following conditions: 2 min denaturation at 94 °C, 37 cycles of 94 °C for 20 s, 58 °C for 50 s, 68 °C for 5–10 min (depending on target size, 1 min/kb), followed by a final extension at 68 °C for 15 min. Positive and negative controls were always executed with each PCR experiment to detect DNA contamination and false positive amplifications. PCR products were checked by agarose gel (1%) electrophoresis. Gel-purified amplification products <3 kb in size were ligated into pGEM-T Easy vectors (Promega), and introduced into *Escherichia coli* (DH5α, Beijing TransGen Biotech, Beijing, China). Followed by ampicillin selection, plasmid DNAs from positive clones were sequenced with M13 primers. Longer PCR products (>3 kb) were directly sequenced with both forward and reverse PCR primers and internal primers by primer walking. All products were sequenced by Life Technologies in Guangzhou, China.

### Sequence assembly, annotation and analysis

SeqMan (DNAStar) was used to assemble the four overlapping nucleotide sequences, which were further confirmed by manually inspection. The protein-coding and rRNA genes were identified using the program ORF Finder (http://www.ncbi.nlm.nih.gov/gorf/gorf.html) and BLAST searches against the GenBank database, respectively. Subsequently, all of these genes were further confirmed by alignment with homologous genes from those of other booklice and lice species. The transfer RNA genes were identified by their cloverleaf secondary structure using ARWEN^46^ with default parameters and tRNAscan-SE 1.21^47^ with Search Mode = EufindtRNA-Cove, Genetic Code = Invertebrate Mito and Cove score cutoff = 0.1. The base composition was analyzed with MEGA 5^48^. Sequences of mt genomes of other booklice and lice were retrieved from GenBank (Table S1).

### Sequence alignment and phylogenetic analysis

Eight species from the Psocoptera and thirteen species from the Phthiraptera were included in our phylogenetic analysis (Table S1). Two true bugs *Alloeorhynchus bakeri* and *Halyomorpha halys* were used as outgroups^12^.

Sequences of all mt protein-coding genes and rRNA genes except *nad4*, *nad4L*, *nad2*, *atp8* were used in phylogenetic analysis. *nad4L* and *atp8* were excluded because they are too short to align among the Psocodean species. *nad4* and *nad2* was excluded because it was not identified in the human pubic louse, *Pthirus pubis*^5^ or in the elephant louse, *Haematomyzus elephantis*^6^. Two alignments were used for phylogenetic analyses: 1) a concatenated nucleotide sequence alignment of nine protein-coding genes and two rRNA genes; 2) a concatenated amino acid sequence alignment of nine protein-coding genes. Nucleotide sequences of all protein-coding genes and rRNA genes were aligned using the default settings in ClustalW as implemented in MEGA 5^48^. Amino acid sequences of PCGs were also aligned in ClustalW. All of the alignments were then imported into the Gblocks server (http://molevol.cmima.csic.es/castresana/Gblocks_server.html) to remove poorly aligned sites^49^. Gblocks server was applied with the ‘codons’, ‘DNA’ and ‘protein’ mode for PCG nucleotide sequences, rRNA sequences and PCG amino acid sequences, respectively, and with all options for a stringent selection were chosen.

Subsequent analyses were performed on the combined dataset using Maximum likelihood (ML) and Bayesian inference (BI). BI was performed using MrBayes 3.2^50^ and ML was performed using RAxML 7.7.1^51^. For ML, the GTRGAMMA model was selected for the concatenated datasets, with 1000 bootstrap replicates. For BI, the best-fitting nucleotides models were chosen using PartitionFinder V1.1.1^52^ as follows: TIM + I + G: *cox1*; GTR + I + G: *atp6*, *cob*, *cox2*, *cox3*, *nad1*, *nad3*, *nad5*; HKY + I + G: *nad6*; TVM + I + G: *rrnL*, *rrnS*; the best-fitting amino acids models were chosen as follows: MtArt + I + G: *cox1*; MtArt + I + G + F: *cox2*, *cox3*, *cob*, *atp6*, *nad1*, *nad3*, *nad5*, and *nad6*. Two independent sets of Markov chains were run, each with one cold and three heated chains for 1 × 10^7^ generations, and every 1000th generation was sampled. Convergence was inferred when a standard deviation of split frequencies <0.01 was completed. Sump and sumt burninfrac was set to 25% and contype was set to allcompat.

### Divergence dating analysis

We performed divergence date analyses based on the combined 11 mt genes dataset of Psocodean (Table S1). The molecular clock was calibrated using three minimum age constraints based on one fossil and two conclusions (100–145 Ma for the split between lice and Liposcelididae, 94–101 Ma for the split between Rhynchophthirina and Anoplura, and the ancestor of three human lice has been stable for at least 7 Ma)^5^,^13^,^14^. Analyses were performed using a relaxed molecular clock model in the Bayesian phylogenetic software BEAST 1.8.0^53^. Rate variation was modeled among branches using uncorrelated lognormal relaxed clocks^53^. A Yule speciation process was used for the tree prior and posterior distributions of parameters, including the tree, were estimated using MCMC sampling^54^. We performed two replicate MCMC runs, with the tree and parameter values sampled every 5000 steps over a total of 50 million generations. A maximum clade credibility tree was obtained using Tree Annotator within the BEAST software package with a burn-in of 1000 trees. Acceptable sample sizes and convergence to the stationary distribution were checked using Tracer 1.5^53^.

## Additional Information

**How to cite this article**: Shi, Y. *et al.* The mitochondrial genome of booklouse, *Liposcelis sculptilis* (Psocoptera: Liposcelididae) and the evolutionary timescale of *Liposcelis.* *Sci. Rep.* **6**, 30660; doi: 10.1038/srep30660 (2016).

## Supplementary Material

Supplementary Information

## Figures and Tables

**Table 1:** PCR primers used for amplification of the mitochondrial genome of *Liposcelis sculptilis.*

Gene	Primer	Primer sequence (5′–3′)	Tm (°C)	Amplicon size (bp)
*cox1*	UEA3	TATAGCATTCCCACGAATAAATAA	58	UEA3-UEA8:1016
*cox1*	UEA8	AAAAATGTTGAGGGAAAAATGTTA		
*cob*	CBF1	TATGTACTACCATGAGGACAAATATC	55	CBF1-CBR1: 481
*cob*	CBR1	ATTACACCTCCTAATTTATTAGGAAT		
*rrnL*	16Sar	CGCCTGTTTAACAAAAACAT	51	16Sar-16Sbr: 513
*rrnL*	16Sbr	CCGGTCTGAACTCAGATCACGT		
*nad5*	N5-F100	GCTATAGCTGCTCCCACCCC	60	N5-F100-N5-R100: 311
*nad5*	N5-R100	ATAAATAAAAGAGCCTTGAATAAAGC		
*cox1*	S1	ATCCAATTCTATTTCAACACCT	62	S1-S2:1987
*nad5*	S2	ATTCATCAACTCTTGTAACAGCCG		
*nad5*	S3	CGGCTGTTACAAGAGTTGATGAAT	62	S3-S4: 4934
*rrnL*	S4	TCTTAGGGTCTTCTCGTCTTTTTAT		
*rrnL*	S5	ATGGGTGGATGCCTTCTAATCTTTA	56	S5-S6: 4387
*cob*	S6	ATGTTAGGAACGGTTCAAGAG		
*cob*	S7	TAGTCTTAGCCCTACCATCAA	57	S7-S8: 3344
*cox1*	S8	AAAATATACACTTCAGGATGACCGA		
*cob*	S9	AGCCAACTATCATACGGTTTTTTTC	61	S9-S10: 9005
*rrnL*	S10	AAACTCGGCAAAATTATGAAGCA		
*rrnL*	S11	CTACCCTGCTCTCTGATTTCAGTTT	60	S11-S12: 5631
*cob*	S12	CTTTTGAGGGGCTACAGTGATTAC		

**Table 2:** The start and stop codons of protein coding gene for *Liposcelis* species.

Gene	*L. decolor*	*L. entomophila*	*L. paeta*	*L. bostrychophila*	*L. sculptilis*
*atp6*	ATG/TAA	ATA/TAA	ATA/TAA	ATA/TAA	ATA/TAA
*atp8*	ATA/TAG	ATA/TAA	ATA/TAG	GTG/TAG	ATA/TAG
*cob*	ATA/TAA	ATA/TAA	ATA/TAA	ATT/TAA	ATA/TAA
*cox1*	TTG/TAA	ATT/TAA	ATA/TAG	ATC/TAA	ATA/TAA
*cox2*	ATT/TAA	ATA/TAA	ATA/TAG	ATA/TAA	ATA/TAA
*cox3*	ATA/TAA	ATA/TAA	TTG/TAA	ATA/TAA	ATG/TAA
*nad1*	ATA/TAA	ATA/TAA	ATT/TAA	ATC/T	ATT/TAA
*nad2*	ATA/TAA	ATA/TAA	ATA/TAA	ATT/TAA	ATT/TAA
*nad3*	ATG/TAA	ATA/TAA	ATA/TAA	ATG/TAG	ATG/TAA
*nad4*	ATA/TAA	ATT/TAA	ATA/TAA	ATC/TAA	ATT/TAA
*nad5*	ATA/TAA	ATT/TAA	ATT/TAA	ATT/TAG	ATA/TAA
*nad6*	ATG/TAA	ATA/TAA	ATA/TAA	ATA/TAA	ATA/TAA
*nad4L*	ATA/TAA		ATA/T	ATT/TAA	
