# Supplementary material for: The mitochondrial genome of booklouse, Liposcelis sculptilis (Psocoptera: Liposcelididae) and the evolutionary timescale of Liposcelis
Source: Sci Rep. 2016 Jul 29;6:30660. doi: 10.1038/srep30660 (PMC4965752; doi:10.1038/srep30660)
Supplement: Supplementary Information [file srep30660-s1.pdf]

Supplementary Section

The mitochondrial genome of booklouse, *Liposcelis sculptilis* (Psocoptera: Liposcelididae) and the evolutionary timescale of *Liposcelis*

Yan Shi, Qing Chu, Dan-Dan Wei, Yuan-Jian Qiu, Feng Shang, Wei Dou, and Jin-Jun Wang*

Key Laboratory of Entomology and Pest Control Engineering, College of Plant Protection, Southwest University, Chongqing 400716, China.

Correspondence: Dr. Jin-Jun Wang, College of Plant Protection, Southwest University, Chongqing 400715, P. R. China. E-mail: wangjinjun@swu.edu.cn; jjwang7008@yahoo.com Tel: (86)-23-68250255; Fax: (86)-23-68251269

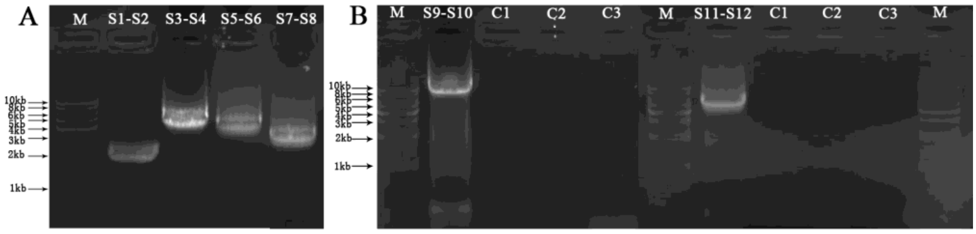

Figure S1. PCR amplification and verification of mitochondrial DNA of *L. sculptilis.* A: Initial Long-PCR amplification of four fragments for the mitochondrial genome of *L. sculptilis*; B: Long-PCR amplification of two fragments to verify the single circular mitochondrial genome in *L. sculptilis*. Lane C1, negative control without the forward primer S9 or S11; lane C2, negative control without the reverse primer S10 or S12; lane C3, negative control without the DNA template. Lane M: 1 kb marker.“S1-S2”, the product of PCR with primers S1 and S2, etc. Primer details are given in Table 1. All gels were run under the same conditions. Full length gels are shown in the following supplementary date section.

Figure S1

Figure S1A

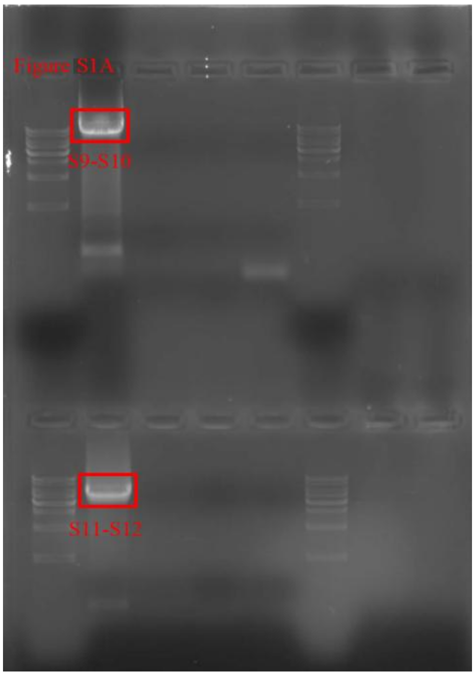

Figure S1B

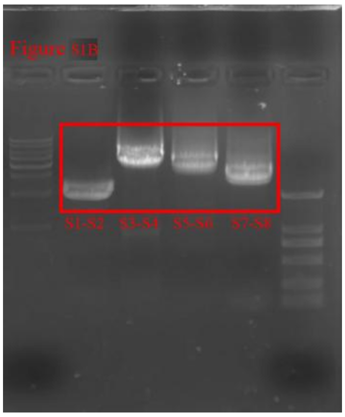

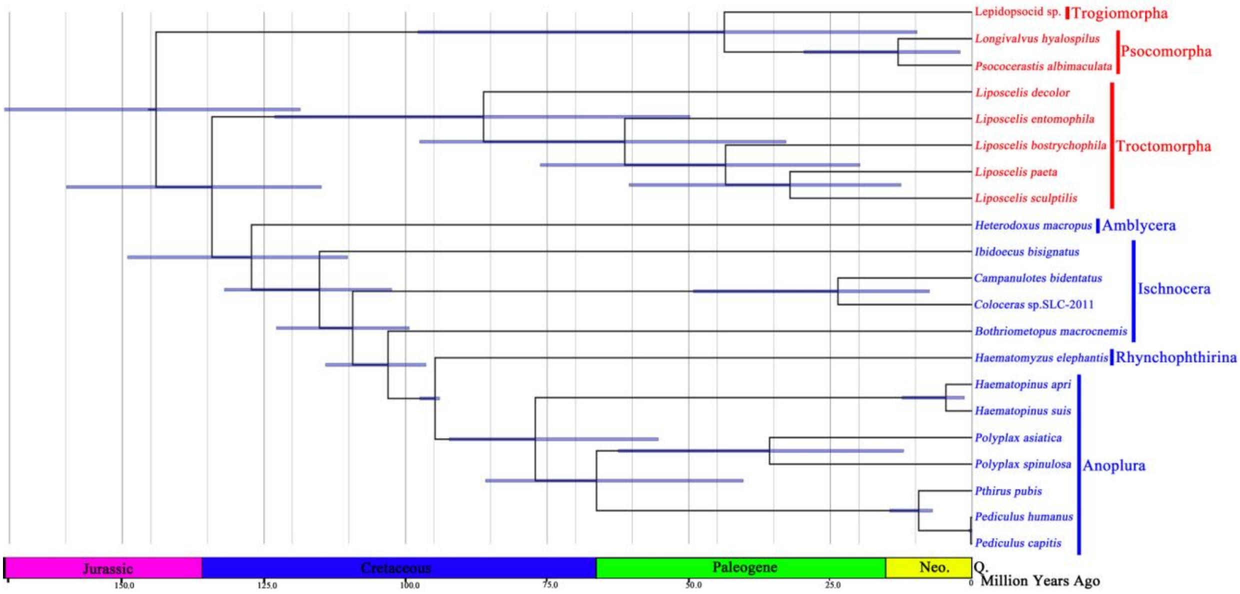

Figure S2. The 95% HPD of Chronogram for the Psocodea. Divergence time chronogram (in millions of years) for Psocodea from analysis of the 11-gene dataset in BEAST. Blue bars indicate 95% highest posterior density (95% HPD) intervals for the node ages.

Table S1. Species of insects used in the phylogenetic analyses in the present study.

Order/suborder	Family	Species	Abb.	GenBank Accession No.
Hemiptera	Nabidae	*Alloeorhynchus bakeri*	Ab	HM235722
	Pentatomidae	*Halyomorpha halys*	Hh	FJ685650
Psocoptera				
Trogiomorpha	Lepidopsocidae	Lepidopsocid sp.	Lsp	NC_004816
Psocomorpha	Psocidae	*Psococerastis albimaculata*	Pa	NC_021400
		Longivalvus hyalospilus	Lh	JQ910986
Troctomorpha	Liposcelidae	*Liposcelis bostrychophila*	Lb	JN645275,JN645276
		Liposcelis decolor	Ld	NC_023839
		Liposcelis entomophila	Le	KF649223,KF649224
		Liposcelis paeta	Lp	KF649225,KF649226
		Liposcelis sculptilis	Ls	Present study
Phthiraptera				
Ischnocera	Philopteridae	*Bothriometopus macrocnemis*	Bm	NC_009983
		Campanulotes bidentatus	Cb	NC_007884
		Ibidoecus bisignatus	Ib	NC_015999
		Coloceras sp. SLC-2011	Cs	JN122000-1
Amblycera	Boopidae	*Heterodoxus macropus*	Hm	AF270939
Rhynchophthirina	Haematomyzidae	*Haematomyzus elephantis*	He	KF933032-41
Anoplura	Haematopinidae	*Haematopinus suis*	Hs	KC814602-10
		Haematopinus apri	Ha	KC814611-19
	Polyplacidae	*Polyplax spinulosa*	Ps	KF647762-72
		Polyplax asiatica	Pas	KF647751-61
	Pthiridae	*Pthirus pubis*	Pp	JQ976018,EU219987-95, HM241895-98
	Pediculidae	*Pediculus capitis*	Pc	JX080388-407
		Pediculus humanus	Ph	FJ499473-90
